# The Metabolic Changes and Immune Profiles in Patients With COVID-19

**DOI:** 10.3389/fimmu.2020.02075

**Published:** 2020-08-28

**Authors:** Bing He, Jun Wang, Yudie Wang, Juan Zhao, Juan Huang, Yu Tian, Cheng Yang, Heng Zhang, Mingxia Zhang, Lixing Gu, Xiaocui Zhou, Jingjiao Zhou

**Affiliations:** ^1^Department of Pediatrics, Renmin Hospital of Wuhan University, Wuhan, China; ^2^Department of Biology and Genetics, College of Life Sciences and Health, Wuhan University of Science and Technology, Wuhan, China; ^3^Institute of Biology and Medicine, The College of Life Sciences and Health, Wuhan University of Science and Technology, Wuhan, China; ^4^Department of Medicine, Hubei Maternal and Child Health Hospital, Wuhan, China

**Keywords:** COVID-19, SARS-CoV-2, acute respiratory distress syndrome (ARDS), metabolic disorders, disseminated intravascular coagulation (DIC), multiple organ dysfunctions (MODS), Inflammatory responses

## Abstract

To explore the metabolic changes and immune profiles in patients with COVID-19, we analyzed the data of patients with mild and severe COVID-19 as well as young children with COVID-19. Of the leukocytes, 47% (IQR, 33–59) were lymphocytes [2.5 × 10^9^/L (IQR, 2.2–3.3)], and monocytes were 0.51 × 10^9^/L (IQR, 0.45–0.57) in young children with COVID-19. In 32 mild COVID-19 patients, circulating monocytes were 0.45 × 10^9^/L (IQR, 0.36–0.64). Twenty-one severe patients had low PO_2_ [57 mmHg (IQR, 50–73)] and SO_2_ [90% (IQR, 86–93)] and high lactate dehydrogenase [580 U/L (IQR, 447–696)], cardiac troponin I [0.07 ng/mL (IQR, 0.02–0.30)], and pro-BNP [498 pg/mL (IQR, 241–1,726)]. Serum D-dimer and FDP were 9.89 mg/L (IQR, 3.62–22.85) and 32.7 mg/L (IQR, 12.8–81.9), and a large number of RBC (46/μL (IQR, 4–242) was presented in urine, a cue of disseminated intravascular coagulation (DIC) in severe patients. Three patients had comorbidity with diabetes, and 18 patients without diabetes also presented high blood glucose [7.4 mmol/L (IQR, 5.9–10.1)]. Fifteen of 21 (71%) severe cases had urine glucose +, and nine of 21 (43%) had urine ketone body +. The increased glucose was partially caused by reduced glucose consumption of cells. Severe cases had extraordinarily low serum uric acid [176 μmol/L (IQR, 131–256)]. In the late stage of COVID-19, severe cases had extremely low CD4^+^ T cells and CD8^+^ T cells, but unusually high neutrophils [6.5 × 10^9^/L (IQR, 4.8–9.6)], procalcitonin [0.27 ng/mL (IQR, 0.14–1.94)], C-reactive protein [66 mg/L (IQR, 25–114)] and an extremely high level of interleukin-6. Four of 21 (19%) severe cases had co-infection with fungi, and two of 21 (9%) severe cases had bacterial infection. Our findings suggest that, severe cases had acute respiratory distress syndrome (ARDS) I–III, and metabolic disorders of glucose, lipid, uric acid, etc., even multiple organ dysfunction (MODS) and DIC. Increased neutrophils and severe inflammatory responses were involved in ARDS, MODS, and DIC. With the dramatical decrease of T-lymphocytes, severe cases were susceptible to co-infect with bacteria and fungi in the late stage of COVID-19. In young children, extremely high lymphocytes and monocytes might be associated with the low morbidity of COVID-19. The significantly increased monocytes might play an important role in the recovery of patients with mild COVID-19.

## Introduction

In December 2019, an unknown viral pneumonia emerged in Wuhan, China, and it then escalated into an unprecedented outbreak (1). Chinese authorities have identified a new type of coronavirus named severe acute respiratory syndrome coronavirus 2 (SARS-CoV-2) (2). On February 11, 2020, the infectious disease caused by this viral strain was officially named COVID-19 (coronavirus disease 2019) by the World Health Organization (WHO) (3). By March 12, COVID-19 had swept into at least 117 countries and killed more than 4,000 people, and WHO officially announced a pandemic of COVID-19 viral disease (4). As of June 21, 2020, COVID-19 cases have been confirmed in 214 countries and territories, and the total was up to 8,708,008, including 461,715 deaths (5).

So far, according to reported patients' data, some remarkable phenomena have been observed. First, only 1% of patients with COVID-19 were infants and young children, and very few young patients have developed severe COVID-19 (6). Leukocytes are the main immune cells to fight against pathogens, and the total leukocyte count is higher in young children than in adults (7). Moreover, the thymus gland of an infant is large and continues to grow throughout childhood. Thus, the thymus produces more than enough matures T-lymphocytes throughout the child's life (8). We explored whether the count and differential of leukocytes in infants and young children are associated with very low morbidity rates of COVID-19.

Second, from the epidemiology and clinical characteristics of COVID-19, 81% of patients were diagnosed as mild cases, and most mild cases can recover from COVID-19 infection (9). So, it could be that specific leukocytes contributed to the recovery of patients with mild COVID-19. Monocytes are important immune sentinel cells critical in the defense against viral infection in the blood. They achieve this via diverse mechanisms that include the detection of viruses, migration into infected tissues, differentiation into macrophages and dendritic cells, and pathogen clearance by phagocytosis and intracellular killing (10, 11). Besides monocytes, the effect of lymphocytes on mild COVID-19 cases is still unclear. In this study, 32 mild patients have been examined to explore the potential roles of monocytes and lymphocytes in the recovery of patients with mild COVID-19.

Third, according to an analysis of nearly 45,000 confirmed cases, 19% of patients with COVID-19 have been identified as severe cases and critically ill cases, involving severe pneumonia and metabolic disorders, developing into acute respiratory distress syndrome (ARDS), multiple organ dysfunctions (MODS), and even septic shock and death (9, 12). Some studies suggested that the immunopathogenesis after viral infection has been linked to the development of the disease into severe cases (13, 14). To explore the potential roles of immuno-pathogenesis in the progress of COVID-19 infection, 21 severe COVID-19 patients have been investigated to explore how the immunopathogenesis was involved in ARDS and metabolic disorders, even MODS, disseminated intravascular coagulation (DIC), and death.

In this study, we investigated mild cases and severe cases infected with SARS-CoV-2, as well as healthy young children and adults. Our multiple comparative analysis showed that not only is leukocyte composition different in healthy groups, these differences can also be found during various stages of SARS-CoV-2 infection. Our study suggests that monocytes, neutrophils, and T-lymphocytes are associated with the onset and progress of COVID-19 infection, and immunopathogenesis was involved in ARDS, metabolic disorders, and MODS in severe cases. This study increases our understanding of the immune responses during COVID-19 infection and provides support to develop novel, feasible, and effective treatments for COVID-19 infection.

## Materials and Methods

### Research Sources: COVID-19 Patients and Healthy Individuals

COVID-19 infection was rapidly endemic in Wuhan, China, in January, 2020. Renmin Hospital of Wuhan University is at the very forefront of the fight against COVID-19. We collected the data of patients with COVID-19, including the clinical records, laboratory results and chest computed tomography (CT) scan images of mild and severe cases in the hospital. For comparison with COVID-19 cases, the data of 35 healthy adults and 31 young children have been collected from the Physical Examination Center of the Hospitals. These healthy individuals have no significant medical condition and were in stable physical condition at that time.

The data of patients with COVID-19 and healthy persons have been all reviewed by a group of professional doctors from the hospitals, including basic features, nucleic acid tests, clinical data, laboratory results, co-infection with other pathogens, CT images, and other primary data. The study design has been approved by the Ethics Committee of the hospital.

### Diagnoses of SARS-CoV-2 Infection

Nasopharyngeal swab samples were collected from patients, and tested as soon as possible to increase the detection rate of SARS-CoV-2. Reverse transcription polymerase chain reaction (RT-PCR) kit (Daan Gene, Shenzhen, China) was used to detect the conserved genes of SARS-CoV-2, such as ORF1ab gene, N gene, and E gene with LightCycler 480 System (Roche, Switzerland). If two or more of these three targeted genes has been detected as positive or one gene has been detected positive in two different samples from the same patient, the result is considered as positive for SARS-CoV-2. Meanwhile, the results can also be analyzed in conjunction with the patient's chest CT images.

### Laboratory Data Analysis of Complete Blood Cell Count, Coagulation Profile, and Metabolic Indicators

Blood samples were collected from patients for laboratory tests. Serum biochemical tests, including aspartate aminotransferase (AST), alanine aminotransferase (ALT), creatine kinase (CK) and lactate dehydrogenase (LDH) were determined with Cobas C501 Testing System (Roche, Germany). Procalcitonin (PCT) and cardiac troponin I (cTn I) were analyzed by CL-2000i Chemiluminescence Immunoassay System (Mindray, Shenzhen, China). Coagulation indicators were detecting with ACL TOP 700 Hemostasis Testing Systems (Werfen, USA). All the blood samples from healthy persons were used for comparison.

### Blood Tests for Immune Cells and Inflammation Factors

To study the count and differential of lymphocytes, the blood samples from COVID-19 patients were stained with CD3, CD4, CD8, CD19, CD16, and CD56 antibodies (BD Multi-test IMK kit, USA) and were analyzed by BD FACSCanto II Flow Cytometer (BD, USA). Th1/Th2 kit (BD, USA) was used to quantitatively measure IL-2, IL-4, IL-10, TNF, and IFN-γ protein levels. To examine the effect of SARS-CoV-2 on the patients' humoral immune function, immunoglobulins (IgM, IgG, IgA, and IgE), complement 3 (C3) and complement 4 (C4) were tested (Siemens Healthineers, USA). C-reactive protein (CRP) and interleukin (IL-6) were measured for COVID-19 patients (Mindray, Shenzhen, China).

### Detections for Co-infection With Other Pathogens

Serum samples of patients were collected and tested for the IgM of respiratory tract pathogens with Pneumoslide IgM kit (Vircell, Spain), including human respiratory syncytial virus, influenza A virus (subtypes H1N1 and H3N2), influenza B virus, parainfluenza virus 1/2/3, metapneumovirus, common coronavirus, Epstein-Barr virus, cytomegalovirus, rhinovirus, adenovirus, and bocavirus, as well as *Legionella pneumophila* serum type I, *Mycoplasma pneumonia*, and *Chlamydia pneumoniae*. Nasopharyngeal secretions were tested for nucleic acids of 13 respiratory pathogens (Health Gene Technologies, Ningbo, China). Sputum culture was performed to identify bacterial and fungal co-infection. The fungal examination was performed with Fungus (1-3)-β-D-Glucan kit (Dynamiker Biotechnology, Tianjin, China) and *Platelia aspergillus* Ag kit (Bio-rad, USA).

### Statistical Analysis

Continuous measurements have been presented as median and interquartile range (IQR) and categorical variables as percentages. For assessing laboratory results, we also assessed whether the measurements were outside the normal range. Unpaired *t*-test with Welch's correction was used for comparison, and *p* < 0.05 and <0.01 were considered statistically significant and highly statistically significant, respectively. GraphPad Prism 8.0.2 (San Diego, CA, USA) and SPSS25.0 (IBM, Armonk, NY, USA) were used for all analyses.

## Results

### The Clinical Characteristics and the Changes of Lymphocytes and Monocytes Presented in Patients With Mild COVID-19

**Figure 1:**
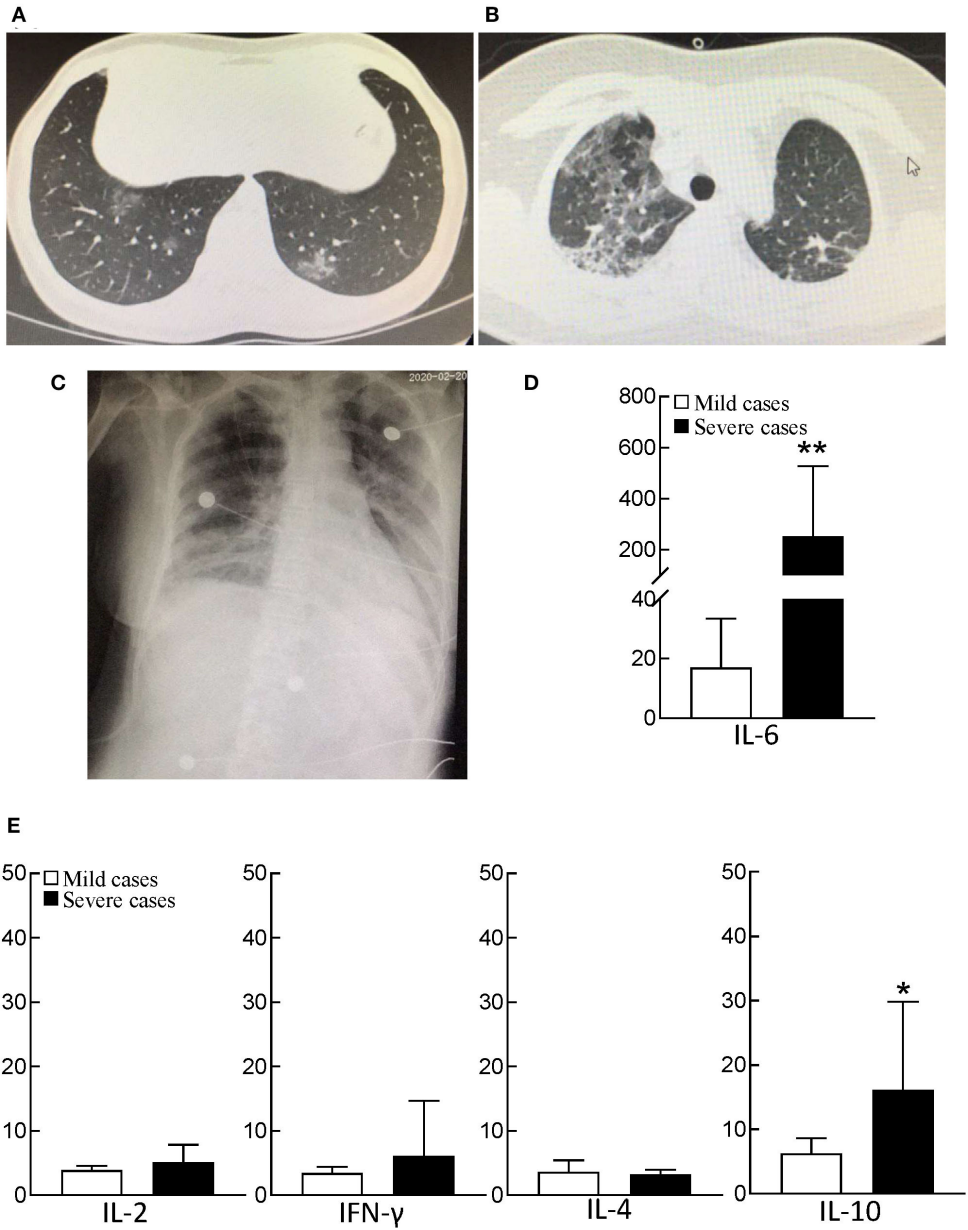
CT and bedside chest X-ray images and serum cytokine concentrations of patients with COVID-19. **(A)** Chest CT image of mild patient showed small patchy, ground glass opacity in the lower lobes of both lungs. **(B)** Chest CT image of severe patient showed critically diffusing, ground glass opacity in the lungs, especially in right lung. **(C)** The critically ill patient's bedside chest X-ray showed the lung texture enhanced and the translucency decreased, and multiple patchy shadows in both lungs. **(D)** serum IL-6 concentration between mild patients (*n* = 32) and severe patients (*n* = 21). The normal range of IL-6 is ≤10 pg/ml. **p < 0.01. **(E)** The analysis of Th1/Th2 cytokine panel between mild patients (*n* = 32) and severe patients (*n* = 21). The normal range of IL-2, IFN-γ, IL-4, and IL-10 are ≤11.4 pg/ml, 18 pg/ml, 12.9 pg/ml, and 5.9 pg/ml, respectively. *p < 0.05.

Patients with fever and/or cough were admitted to hospital after February 1, 2020. Chest CT images indicated multiple patchy, ground-glass opacity in the lungs (Figure 1A). Thirty-two patients were further diagnosed as infected with SARS-CoV-2 by real-time RT-PCR. There were 17 men and 15 women, and the median age of these mild cases was 42. The clinical characteristics of mild patients were presented in Supplementary Table S1.

Compared with healthy adults, the count of leukocytes and neutrophils in mild COVID-19 patients did not increase, but the median percentage and count of lymphocytes were 26% (IQR, 19–34) and 1.2 × 10^9^/L (IQR, 1.1–1.6), respectively, which were significantly less than those of healthy adults, 34% (IQR, 29–39) and 2.0 × 10^9^/L (IQR, 1.8–2.5), respectively (*p* < 0.001). Interestingly, the median percentage and count of monocytes were 8.2% (IQR, 7.1–9.2) and 0.45 × 10^9^/L (IQR, 0.36–0.64), which were significantly higher than those of healthy adults 6.3% (IQR, 5.5–7.1) and 0.39 × 10^9^/L (IQR, 0.35–0.42) (*p* ≤ 0.001) (Table 1). The significantly increased number of monocytes could play an important role in the recovery of patients with mild COVID-19.

**Table 1:** Leukocyte count and differential of patients with COVID-19 and healthy adults.

		**Medium (IQR)**					
	**Normal range**	**Healthy adults**	**Mild Covid-19 patients**	**Severe Covid-19 patients**	***P*-value^**a**^**	***P*-value^**b**^**	***P*-value^**c**^**
		**(*n* = 35)**	**(*n* = 32)**	**(*n* = 21)**			
WBC, ×10^9^/L	3.5–9.5	6.2 (5.7–6.7)	4.7 (4.1–6.7)	7.6 (5.5–11.3)	0.3651	<0.001	<0.001
Lymphcyte, %	20–50	34 (29–39)	26 (19–34)	7 (4–10)	<0.001	<0.001	<0.001
Monocyte, %	3–10	6.3 (5.5–7.1)	8.2 (7.1–9.2)	4.5 (3.1–6.2)	<0.001	<0.001	<0.001
Neutrophil, %	40–75	56 (52–63)	64 (56–71)	88 (84–92)	0.004	<0.001	<0.001
Eosinophil, %	0.4–8.0	1.7 (1.0–2.4)	0.7 (0–2.6)	0 (0–0)	0.486	<0.001	<0.001
Basophil, %	0–1.0	0.7 (0.4–0.8)	0.3 (0.2–0.5)	0.1 (0–0.2)	0.001	<0.001	<0.001
Lymphcyte, ×10^9^/L	1.1–3.2	2.0 (1.8–2.5)	1.2 (1.1–1.6)	0.5 (0.3–0.8)	<0.001	<0.001	<0.001
Monocyte, ×10^9^/L	0.1–0.6	0.39 (0.35–0.42)	0.45 (0.36–0.64)	0.37 (0.21–0.51)	0.001	0.289	0.023
Neutrophil, ×10^9^/L	1.8–6.3	3.3 (3.1–4.3)	3.2 (2.3–4.6)	6.5 (4.8–9.6)	0.521	<0.001	<0.001
Eosinophil, ×10^9^/L	0.02–0.52	0.11 (0.05–0.15)	0.03 (0–0.12)	0 (0–0)	0.159	<0.001	<0.001
Basophil, ×10^9^/L	0–0.06	0.03 (0.03–0.05)	0.02 (0.01–0.03)	0.01 (0–0.02)	0.001	<0.001	0.019

*COVID-19, coronavirus disease 2019; IQR, interquartile range; WBC, white blood cell*.

a *P-values indicate differences between mild COVID-19 patients and healthy adults. P < 0.05 was considered statistically significant*.

b *P-values indicate differences between severe COVID-19 patients and healthy adults. P < 0.05 was considered statistically significant*.

c *P-values indicate differences between mild and severe COVID-19 patients. P < 0.05 was considered statistically significant*.

### The Exceptionally High Lymphocytes and Monocytes Might Be Associated With Low Morbidity of COVID-19 in Young Children

To investigate why infants and young children have low morbidity of COVID-19, we analyzed the clinical characteristics of young children with COVID-19, and collected the data of circulating leukocytes of young children with/without COVID-19. Comparative analyses showed that young children have much higher leukocyte counts [6.9 × 10^9^/L (IQR, 6.1–8.1)] than adults. Of note, 51% (IQR, 42–58) of leukocytes are lymphocytes [3.4 × 10^9^/L, (IQR, 2.5–4.6)] in young children. The median count of monocytes in young children is 0.46 × 10^9^/L (IQR, 0.41–0.67), which is much higher than that of adults [0.39 × 10^9^/L (IQR, 0.35–0.42)] (*p* = 0.001). Lymphocytes of young children with COVID-19 was a little lower than those of healthy children, but remained at a high level [2.5 × 10^9^/L (IQR, 2.2–3.3)]. Young children with COVID-19 had a high level of monocytes [0.51 × 10^9^/L (IQR, 0.45–0.57)] as well (Table 2). Such a high number of lymphocytes and monocytes has benefit to fight against SARS-CoV-2, which might be associated with the low morbidity of COVID-19 in young children.

**Table 2:** The clinical characteristics and leukocyte count and differential of young children with COVID-19.

	**Median (IQR)**		
	**Healthy young children**	**Healthy children with COVID-19**	***P*-value**
	**(*n* = 31)**	**(*n* = 16)**	
**Age (Y)**	4 (2–6)	6 (3–7)	
**Gender (M/F)**	16 (52%)/15 (48%)	10 (62%)/6 (38%)	
**Mild case/Severe case**	NA	1 (6%)/15 (94%)	
**Signs and symptoms at admission**			
Fever	NA	6 (38%)	
Cough	NA	12 (75%)	
Sputum	NA	0	
Shortness of breath	NA	1 (6%)	
Diarrhea	NA	0	
**Treatment**			
Antibiotic treatment	NA	10 (62%)	
Antiviral treatment	NA	10 (62%)	
hormone therapy	NA	1 (6%)	
**Ventilation**			
Non-invasive (face mask, etc)	NA	1 (6%)	
Mechanical ventilation	NA	0	
**Discharged**	NA	16 (100%)	
**WBC**, **×10**^**9**^**/L**	6.9 (6.1–8.1)	5.6 (5.2–6.2)	0.007
Lymphcyte, %	51 (42–58)	47 (33–59)	0.239
Monocyte,%	6.7 (5.5–8.0)	8.7 (7.3–11.3)	0.027
Neutrophil, %	38 (33–46)	40 (26–65)	0.282
Eosinophil, %	2.1 (0.9–4.5)	2.6 (1.3–5.2)	0.646
Basophil,%	0.5 (0.2–0.7)	0.4 (0.3–0.5)	0.741
Lymphcyte, ×10^9^/L	3.4 (2.5–4.6)	2.5 (2.2–3.3)	0.008
Monocyte, ×10^9^/L	0.46 (0.41–0.67)	0.51 (0.45–0.57)	0.696
Neutrophil, ×10^9^/L	2.6 (2.1–3.0)	3.0 (2.0–4.1)	0.286
Eosinophil, ×10^9^/L	0.15 (0.06–0.32)	0.15 (0.04–0.29)	0.572
Basophil, ×10^9^/L	0.03 (0.02–0.05)	0.02 (0.01–0.04)	0.673

*P-values indicate differences between young children with COVID-19 and healthy young children. P < 0.05 was considered statistically significant*.

### Patients With Severe COVID-19 Suffered From Severe Acute Respiratory Syndrome (ARDS, I-III)

We collected and compared the data of 21 severe cases and 32 mild cases. Chest CT images of severe cases indicated that there was critically diffuse ground-glass opacity in both lungs. A representative CT image is presented in Figure 1B. In bedside chest X-ray results of the critically ill patients, the translucency of both lungs was diffusely decreased, and a large area of patchy shadow appeared with uneven density. Tracheal intubation can be observed in the trachea and the heart shadow outline (Figure 1C). The clinical characteristics of severe patients were presented in Supplementary Table S1.

These CT and X-ray images showed that the primary and most significant changes were in the lower respiratory tract of patients with severe COVID-19. Among the respiratory indicators we measured, severe cases had lower partial pressure of oxygen (PO_2_) and oxygen saturation (SO_2_), 57 mmHg (IQR, 50–73) and 90% (IQR, 86–93), respectively, and suffered from different degrees of ARDS, I to III (Table 3).

**Table 3:** The metabolic disorders and multi-organ dysfunctions in severe patients with COVID-19.

		**Median (IQR)**		
	**Normal range**	**Mild patients (*n* = 32)**	**Severe patients (*n* = 21)**	***P-*value^**a**^**
P02, mm Hg	80–100	85 (82–115)	57 (50–73)	0.003
S02, %	95–98	97 (95–98)	90 (86–93)	<0.001
PC02, mm Hg	35–45	43 (39–47)	37 (33–40)	0.08
PH	7.35–7.45	7.39 (7.34–7.44)	7.46 (7.42–7.50)	0.04
BE, mmol/L	−3–3	2 (−1.3–4.3)	3.2 (−0.3–5)	0.28
cTnl, ng/ml	0–0.04	<0.01	0.07 (0.02–0.30)	
Mb, μg/L	0–110	29 (20–35)	54 (40–84)	<0.001
CK, U/L	50–310	44 (31–82)	92 (50–153)	0.006
CK-MB, ng/ml	0–5	0.6 (0.5–0.8)	1.3 (0.9–2.5)	<0.001
LOH, U/L	120–250	197 (170–229)	580 (447–696)	<0.001
Pro-BNP, pg/ml	0–450	21 (8–97)	498 (241–1,726)	0.001
ALT, U/L	9–50	20 (11–33)	23 (17–44)	0.228
AST, U/L	15–35	18 (15–27)	33 (26–64)	0.007
ALP, U/L	45–125	57 (46–71)	73 (54–98)	0.001
y-GT, U/L	7–45	24 (14–42)	45 (31–69)	<0.001
TP, g/L	65–85	65 (62–67)	61 (57–65)	0.006
ALB, g/L	40–55	42 (37–44)	32 (29–34)	<0.001
A/G	1.2–2.4	1.7 (1.5–2.1)	1.1 (0.9–1.3)	<0.001
TBIL, μmol/L	0–23	8.3 (6.5–11.2)	13 (8.5–17.6)	0.027
DBIL, μmol/L	0–8	2.8 (2.2–4.2)	5.1 (3.5–7.9)	<0.001
Glucose, mmol/L	3.9–6.1	5.2 (4.9–6.3)	7.4 (5.9–10.1)	<0.001
Uric acid, μmol/L	208–428	275 (218–324)	176 (131–256)	<0.001
Cr, μmol/L	57–97	57 (49–69)	56 (50–66)	0.377
Urea, mmol/L	3.1–8	3.8 (3.3–4.3)	7.8 (5.9–9.1)	<0.001
GFR, mL/min	>90	119 (112–122)	101 (93–109)	<0.001
Na+, mmol/L	135–145	141 (139–143)	141 (138–145)	0.598
K+, mmol/L	3.5–5.5	3.9 (3.6–4.2)	3.7 (3.4–4.1)	0.461
Cl-, mmol/L	99–110	105 (103–107)	105 (101–107)	0.818
Ca2+, mmol/L	2.11–2.52	2.19 (2.11–2.25)	1.97 (1.89–2.05)	<0.001
Mg2+, mmol/L	0.75–1.02	0.84 (0.80–0.89)	0.86 (0.80–0.93)	0.642
D-dimer, mg/L	0–0.55	0.38 (0. 19–0.79)	9.89 (3.62–22.85)	<0.001
FDP, mg/L	0–5	0.9 (0.3–2.8)	32.7 (12.8–81.9)	<0.001
PT, s	9–13	12 (12–13)	13 (12–13)	0.132
PS, %	75–135	83 (74−87)	77 (68–87)	0.234
APTT, s	25–31.3	29.1 (26.6–30.5)	27.8 (25.9–33.6)	0.242
FIB, g/L	2–4	3.6 (2.9–5.0)	3.3 (1.5–4.4)	0.07
AT-III,%	80–120	92 (86–100)	78 (71–85)	<0.001
**Urine**				
Urine glucose (+)	Negative	NA	15/21 (71%)	
Urine ketone body (+)	Negative	NA	9/21 (43%)	

*PO_2_, partial pressure of oxygen; SO_2_ (%), oxygen saturation; PCO_2_, partial pressure of carbon dioxide; BE, Base Excess; cTnI, high-sensitivity cardiac troponin I; Mb, myoglobin; CK, creatine kinase; CK-MB, creatine kinase isoenzyme; LDH, Lactate dehydrogenase; Pro-BNP, Pro-Brain-type natriuretic peptide; ALT, alanine aminotransferase; AST, aspartate aminotransferase; ALP, alkaline phosphatase; γ-GT, γ-glutamyl transpeptidase; TP, total protein; ALB, albumin; A/G, albumin-globulin ratio; TBIL, total bilirubin; DBIL, direct bilirubin; Cr, creatinine; GFR, glomerular filtration rate; Na^+^, sodium; K^+^, potassium; Cl^−^, chloride; Ca^2+^, calcium; Mg^2+^, magnesium; FDP, fibrinogen degradation products; PT, prothrombin time; PS, prothrombin time activity; APTT, activated partial thromboplastin time; FIB, fibrinogen; AT-III, antithrombin III*.

*P-values indicate differences between mild patients and severe patients. P < 0.05 was considered statistically significant*.

### Severe COVID-19 Cases Had Metabolic Disorders, MODS, and Coagulation Disorders

Several cardiac parameters increased sharply, LDH [580 U/L (IQR, 447–696)], cardiac troponin I (cTnI) [0.07 ng/mL (IQR, 0.02–0.30)], as well as and pro-B-type natriuretic peptide (pro-BNP) [498 pg/mL (IQR, 241–1,726)], which indicated the heart function disorder, even heart failure in patients with severe COVID-19. Comparing the indicators of liver and kidney functions with those of mild cases, severe cases had higher AST [33 U/L (IQR, 26–64)] and glutamyltransferase (γ-GT) [45 U/L (IQR, 31–69)] and lower albumin (ALB) [32 g/L (IQR, 29–34)] and albumin/globulin ratio [1.1 (IQR, 0.9–1.3)] (*p* < 0.01); they also had higher urea [7.8 mmol/L (IQR, 5.9–9.1)] and lower Ca^2+^ (1.97 mmol/L (IQR, 1.89–2.05). Severe patients also had less of fibrinogen (FIB) [3.3 g/L (IQR 1.5–4.4)] and antithrombin III [78% (IQR, 71–85)]. For healthy people, the reference range of D-dimer is 0–0.55 mg/L, and the range for fibrin degradation product (FDP) is 0–5 mg/L. The severe cases had exceptionally high amounts of D-dimer and FDP, 9.89 mg/L (IQR, 3.62–22.85) and 32.7 mg/L (IQR, 12.8–81.9), respectively (Table 3). A high count of red blood cells (RBC) [46/μL (IQR, 4–242)] was presented in the urine of patients with severe COVID-19 (Table 4).

**Table 4:** Immune and inflammatory profiles of patients with COVID-19.

		**Median (IQR)**		
	**Normal**	**Mild patients**	**Severe patients**	***P*-value**
	**range**	**(*n* = 32)**	**(*n* = 21)**	
**Lymphocytes**				
CD3+, %	56–86	69 (66–77)	56 (49–66)	<0.001
CD3+, /μL	723–2,737	794 (586–1,112)	221 (168−414)	<0.001
CD4+, %	33–58	40 (36–46)	38 (27–46)	0.043
CD4+, /μL	404–1,612	433 (318−651)	146 (107–277)	<0.001
CD8+, %	13–39	26 (22–32)	15 (9–24)	<0.001
CD8+, /μL	220–1,129	297 (230–388)	59 (33–109)	<0.001
CD4+/CD8+	0.9–2.0	1.45 (1.24–1.80)	2.38 (1.62–4.63)	<0.001
CD19+, %	5–22	13 (9–19)	23 (13–33)	<0.001
CD19+, /μL	80–616	125 (88−237)	91 (54–181)	0.123
CD16+CD56+, %	5–26	12 (9–18)	16 (10–19)	0.098
CD016+ CD56+, /μL	84–724	128 (87–213)	63 (26–109)	0.061
**Humoral immunity**				
Serum gobulin, g/L	20–40	22 (21–26)	30 (26–33)	<0.001
lgM, g/L	0.4–2.3	1.0 (0.8−1.2)	0.7 (0.6–0.8)	0.027
lgG, g/L	7.0–16.0	11.2 (10.2–16.0)	16.6 (13.7–21.4)	0.016
lgA, g/L	0.7–4.0	2.4 (1.9–3.0)	2.3 (1.5–2.7)	0.263
lgE, IU/ml	<100	92 (55–170)	112 (75–191)	0.339
C3, g/L	0.9–1.8	1.1 (0.9–1.2)	0.9 (0.7–1.0)	0.002
C4, g/L	0.1–0.4	0.3 (0.2–0.3)	0.2 (0.1–0.2)	0.062
**Inflammatory responses**				
CRP, mg/L	<10	24 (11–51)	66 (25–114)	0.003
PCT, ng/ml	<0.1	0.05 (0.03–0.07)	0.27 (0.14–1.94)	0.02
**Urine**				
RBC, /μL	0–10	NA	46 (4–242)	
WBC, /μL	0–12	NA	18 (9–46)	

*IgM, Immunoglobulin M; IgG, Immunoglobulin G; IgA, Immunoglobulin A; IgE, Immunoglobulin E; C3, complement C3; C4, complement C4; CRP, C-reactive protein; PCT, procalcitonin*.

*P-values indicate differences between mild and severe patients. P < 0.05 was considered statistically significant*.

Increased glucose and low uric acid in blood should be noted here. The level of blood glucose was 5.2 mmol/L (IQR, 4.9–6.3) in 32 mild cases. Three of 21 (14%) severe cases had comorbidity with diabetes mellitus. Eighteen severe cases without comorbidity of diabetes also had high blood glucose [7.4 mmol/L (IQR, 5.9–10.1)]. Critically ill patients had extremely high levels of blood glucose [8.9 mmol/L (IQR, 6.8–12.9)]. Meanwhile, 15 of 21 (71%) severe cases had positive urine glucose +, and 9 of 21 (43%) severe cases had positive urine ketone body +. Additionally, serum uric acid was 275 μmol/L (IQR, 218–324) in mild cases, whereas an extraordinarily low level of serum uric acid [176 μmol/L (IQR, 131–256)] was found in severe cases (Table 3).

### Severe Cases Had a Dramatical Decrease of T-lymphocytes and a Potentially High Risk of Co-infection

The total of leukocytes was 7.6 × 10^9^/L (IQR, 5.5–11.3) in the peripheral blood of severe cases, which were much more than those in mild cases. Compared with mild cases, severe cases had low levels of monocytes [0.37 × 10^9^/L (IQR, 0.21–0.51)]. However, the percentage and count of lymphocytes in severe cases were only 7% (IQR, 4–10) and 0.5 × 10^9^/L (IQR, 0.3–0.8) respectively, which were significantly lower than those in mild cases (Table 1).

The subsets of lymphocytes were examined by flow cytometry, including natural killer (NK) cells (CD16^+^CD56^+^), B cells (CD19^+^), and T cells (CD3^+^). The results showed that severe cases had NK cells [63/μL (IQR, 26–109)] and B cells [91/μL (IQR, 54–181)], which was not a significant difference from the mild cases (*p* > 0.05). In addition, the functions of B cells and complements were tested, including IgM, IgG, IgA, IgE, C3, and C4, for both mild and severe COVID-19 cases. For severe cases, the values of IgM, C3, and C4 were slightly lower than those in mild cases, but these values were still within the normal range. However, compared with mild cases, severe cases had much lower levels of CD4^+^ T cells and CD8^+^ T cells, 146/μL [IQR, 107–277] and 59/μL (IQR, 33–109), respectively. The decrease of CD8^+^ T cells was much more than that of CD4^+^ T cells, and the ratio of CD4^+^ T cells/CD8^+^ T cells increased by 2.38 (IQR, 1.62–4.63) (Table 4). Further examination of Th1/Th2 cytokines also indicated that severe patients had normal levels of IL-2, and IFN-γ, as well as IL-4 in peripheral blood, but the level of IL-10 in severe patients was 4 times higher than normal (Figure 1E).

In this study, the clinical course of severe cases was over 3 weeks, and severe cases had a potentially high risk of co-infection with other pathogens due to critical exhaustion of CD4^+^ and CD8^+^ T cells. The respiratory tract pathogens were tested in severe cases, including 10 viruses, *Legionella pneumophila, Mycoplasma pneumoniae*, and *Chlamydia pneumoniae*, which were all negative. The fungal examinations, G assay and GM assay, were also performed in severe cases. The results of bacterial and fungal examinations indicated that four of 21 (19%) severe cases had co-infection with fungi, and two of 21 (9%) severe cases had co-infection with bacteria. A high number of white blood cells (WBC) [18/μL (IQR, 9–46)] was found in the urine of severe cases (Table 4).

### Exceptionally High Neutrophils and Severe Inflammatory Responses Might Be Involved in ARDS, MODS and Coagulation Disorders

Further examinations showed that the median PCT was 0.27 ng/mL (IQR, 0.14–1.94) in severe cases, a cue of potential sepsis/septic shock. Among the inflammatory factors tested in severe cases, the median of CRP was 66 mg/L (IQR, 25–114), which was much higher than those in mild cases (Table 4). IL-6 slightly increased in mild cases, but exceptionally high level of IL-6 presented in severe cases, even 40 times higher than normal in some critically ill cases (Figure 1D). The release of the inflammatory factors triggered by SARS-CoV-2 replication and/or co-infection with bacteria and fungi, played important roles in the progress of COVID-19 infection.

In the late stage of the disease in severe COVID-19 cases, 88% (IQR, 84–92) of leukocytes were neutrophils [6.5 × 10^9^/L (IQR, 4.8–9.6)] (Table 1). Previous studies showed that largely number of neutrophils triggered inflammatory responses and caused excessive organ injury in acute inflammatory disease, such as sepsis (15, 16). Exceptionally high neutrophil numbers might be involved in severe inflammatory responses and might be associated with ARDS, MODS, and even sepsis/septic shock, DIC, and death during the late stage of severe COVID-19 infection.

## Discussion

In this study, we first analyzed the clinical features and leukocyte differential of mild COVID-19 patients admitted to the hospital after February 1, 2020. Thirty-two mild cases, with a median age of 42 years, had recovered from COVID-19 infection. Our data showed that compared with healthy adults, patients with mild COVID-19 had lower lymphocytes in the acute stage, which was consistent with previous studies (12). However, mild COVID-19 cases had high counts of circulating monocytes [0.45 × 10^9^/L (IQR, 0.36–0.64)]. In addition, mild patients had normal level of IL-4 and IL-10 in peripheral blood, but they had a 1–2-fold increase of IL-6. Monocytes/macrophages play very important roles in fighting against invading foreign viruses. Literature from the past 30 years has emphasized links among IL-6 and innate immune response, such as mononuclear phagocytes (10, 11, 17). For patients with mild COVID-19, a high monocyte count and slight increase of IL-6 might be helpful for eradicating the SARS-CoV-2 infection and were associated with recovery from COVID-19.

Based on the epidemiology and clinical characteristics of COVID-19, young children under six have the lowest morbidity rate, and very few young children with COVID-19 develop severe cases (6, 18). According to our comparative analysis, young children under six have highly circulating monocytes, and 51% (IQR, 42–58) of leukocytes are lymphocytes [3.4 × 10^9^/L (IQR, 2.5–4.6)], including B-lymphocytes and T-lymphocytes. Lymphocytes of young children with COVID-19 was a little lower than those of healthy children, but remained at a high level [2.5 × 10^9^/L (IQR, 2.2–3.3)]. Young children with COVID-19 had a high level of monocytes [0.51 × 10^9^/L (0.45–0.57)] as well. The intricate process of T-lymphocyte development in the thymus is essential in the formation and maintenance of the peripheral T-lymphocytes. The thymus of a young child is big, and has the function of maintaining the large amounts of T-lymphocytes in the peripheral blood (19, 20). Extremely high levels of circulating lymphocytes and monocytes would benefit to fight against SARS-CoV-2 infection, which might be associated with the low morbidity of COVID-19 in young children.

To explore the metabolic changes and immune responses in the progress of COVID-19 cases, we investigated 21 patients with severe COVID-19 infection. The median age of these patients was 57, and the clinical course was more than 3 weeks. CT scan images showed multiple patchy ground-glass shadows in the left and right lungs. Bedside chest radiography of critically ill patients indicated that the brightness of both lungs was decreased and multiple patchy shadows were observed. These clinical characteristics of severe cases are very similar to those reported in previous studies (21, 22). The 21 severe COVID-19 cases had ARDS I to III, and had extremely high levels of cTnI, LDH, and pro-BNP, a marker of severe cardiac dysfunction and even heart failure. Besides that, an extraordinarily low level of serum uric acid [176 μmol/L (131–256)] was found in severe cases. Uric acid is synthesized mainly in the liver and other tissues, which usually dissolves in the blood, and is removed from the body through urine. The extraordinarily low level of serum uric acid might indicate that potential liver and/or rental metabolism dysregulated in severe patients.

Among 21 severe cases, three patients had the comorbidity of diabetes, and other patients also had very high blood glucose [7.4 mmol/L (IQR, 5.9–10.1)]. Meanwhile, 15 out of 21 (71%) severe patients has positive of urine glucose (+), and nine out of 21 (43%) severe patients had positive of urine ketone body (+). The increased glucose of blood and urine was partially caused by the reduced glucose consumption of cells in severe patients. We need to pay attention to the high risks of metabolic syndromes mediated by high blood glucose, high urine glucose and urine ketone bodies. Dramatically high level of D-dimers [9.89 mg/L (IQR, 3.62–22.85)] and FDP [32.7 mg/L (IQR, 12.8–81.9)] were found in severe patients. A large amount of RBC [46/μL (IQR, 4–242)] was in urine of severe patients. These results showed that severe coagulation disorders, even DIC, occurred in these severe cases.

We further investigated immune responses in patients with severe COVID-19. First, different subpopulations of lymphocytes were investigated. The percentage and count of B cells and NK cells did not obviously change, which is consistent with the results from a previous report (23). The results of IgM/IgG/IgA/IgE, C3 and C4 also indicated that B cells and complements held normal functions. However, compared with mild cases, severe COVID-19 cases had lower levels of CD4^+^ T cells [146/μL (IQR, 107–277)] and an even more significant reduction in CD8^+^ T cells [only 59/μL (IQR, 33–109)], which has a sharper drop than CD4^+^ T cell. We further analyzed Th1/Th2 panel, in severe patients, Th1 cytokines (IL-2 and IFN-γ) were in the normal range, but IL-10, one of Th2 cytokines, was about four times higher than normal. Previous studies presented that in severe patients, CD4^+^ T cells and CD8^+^ T cells highly expressed the exhaustion markers, including NKG2A, PD-1, and Tim-3 (24, 25). The dramatical decrease and functional exhaustion of CD4^+^ T cells and CD8^+^ T cells represents an important immunological characteristic of severe COVID-19 infection. Following the exhaustion of T cells, severe cases had high potential for co-infection with other pathogens. In this study, 4 of 21 (19%) severe patients had co-infection with fungi, and two of 21 (9%) severe patients had bacterial co-infection.

Twenty-one severe cases had a high level of PCT and CRP, 0.27 ng/mL (IQR, 0.14–1.94) and 66 mg/L (IQR, 25–114), respectively. IL-6 was much higher than normal in severe cases, even 40 times higher than normal in some critically ill cases. With SARS-CoV-2 replication and/or co-infection with bacteria and fungi, severe inflammatory responses played important roles in the progress of severe COVID-19 infection. In the late stage of severe COVID-19, 88% (IQR, 84–92) of leukocytes were neutrophils [6.5 × 10^9^/L (IQR, 4.8–9.6). A high number of WBC [18/μL (IQR, 9–46)] was presented in urine of severe patients. Previous studies suggest that, in sepsis, a large number of neutrophil and the formation of neutrophil extracellular traps (NET) triggered severe inflammatory responses and excessive tissue damage (15, 16, 26). The significant increase in neutrophils might be involved in severe inflammatory responses and MODS, even DIC and death in severe COVID-19 patients. Additionally, uric acid is the predominant anti-oxidant molecule in the plasma and respiratory tract, and is necessary for induction of type 2 immune responses. Uric acid plays a pivotal role in protecting against pathogen infections and autoimmune diseases (27, 28). Whether the decrease of serum uric acid is associated with the inflammatory responses in severe COVID-19 cases need to be explored.

There are several limitations to this study. First, we investigated 16 young children with COVID-19 and 53 adult cases, including 32 mild cases and 21 severe cases. More cases will need to be collected for comparative analysis of the difference between severe and critically ill patients. Second, more inflammatory cytokines and chemokines will be analyzed for severe and critically ill patients and will be further evaluated for inflammatory storm mediated ARDS, DIC, MODS, and coagulation disorders. Third, the mechanisms by which SARS-CoV-2 infection causes the reduction and functional exhaustion of CD4^+^ T cells and CD8^+^ T cells are still unclear. *In-vitro* and *in-vivo* experiments need to be performed to explore the mechanisms of T cell exhaustion.

In summary, our findings suggest that extremely high level of lymphocytes and monocytes could help hamper SARS-CoV-2 replication, which might be associated with the low morbidity of COVID-19 in infants and young children. A high number of monocytes would be helpful for removing SARS-CoV-2 and play an important role in the recovery of patients with mild COVID-19. In the late stage of the disease, severe cases suffered from ARDS, metabolic disorders, MODS and coagulation disorders. With dramatical decrease of CD4^+^ T cells and CD8^+^ T cells, extraordinarily increased neutrophils and severe inflammatory responses are involved in ARDS, MODS, and coagulation disorders and can even lead to DIC and death in severe cases. Whether the decrease of serum uric acid is associated with the inflammatory responses in severe COVID-19 cases needs to be further explored. These findings can not only greatly improve our understanding of metabolic and immunological characteristics, but also provide a mechanistic basis for the prevention and treatment of COVID-19 infection.

## Data Availability Statement

All datasets generated for this study are included in the article/Supplementary Material.

## Ethics Statement

The studies involving human participants were reviewed and approved by Renmin hospital of Wuhan University. Written informed consent for participation was not required for this study in accordance with the national legislation and the institutional requirements.

## Author Contributions

JZho, XZ, and BH developed the concept and designed this study. JZho, XZ, BH, JW, LG, YW, JH, and JZha contributed the acquisition, analysis, and interpretation of data. JZho, BH, JW, YW, and LG contributed drafting of the manuscript and critical revision of the manuscript for important intellectual content. JW, YW, JZha, and CY conducted the statistical analysis. YT, HZ, JW, CY, JZha, JH, and MZ performed the administrative, technical, and material support duties. All authors contributed to the article and approved the submitted version.

## Conflict of Interest

The authors declare that the research was conducted in the absence of any commercial or financial relationships that could be construed as a potential conflict of interest.
