# Supplementary material for: The Metabolic Changes and Immune Profiles in Patients With COVID-19
Source: Front Immunol. 2020 Aug 28;11:2075. doi: 10.3389/fimmu.2020.02075 (PMC7485144; doi:10.3389/fimmu.2020.02075)
Supplement: Supplementary file 1 [file Table_1.pdf]

Supplementary Table S1 Clinical characteristics of patients with COVID-19

	Mild patients (n=32)	Severe patients (n=21)
**Age (Y)**	42 (27-55)	57 (49-68)
**Gender (M/F)**	15/17	13/8
**Signs and symptoms at admission**		
Fever	20/32 (63%)	12/21 (57%)
Cough	16/32 (50%)	10/21 (48%)
Sputum	2/32 (6%)	3/21 (14%)
Shortness of breath	2/32 (6%)	4/21 (19%)
Diarrhea	No	1/21 (5%)
**Comorbidities**		
Cardiovascular and cerebrovascular	2/32 (6%)	6/21 (28%)
Endocrine system diseases （Diabetes)	No	3/21 (14%)
Digestive system disease	No	No
Respiratory system diseases	No	1/21 (5%)
Malignant tumor	No	1/21 (5%)
Nervous system diseases	No	1/21 (5%)
Chronic kidney disease	1/32 (3%)	1/21 (5%)
Chronic liver disease	No	1/21 (5%)
COPD	No	No
**Treatment**		
Antibiotic treatment	5/32 (16%)	12/21 (57%)
Antiviral treatment	22/32 (69%)	21/21 (100%)
hormone therapy	No	8/21 (38%)
**Ventilation**		
Non-invasive(face mask, etc)	No	14/21 (67%)
Mechanical ventilation	No	7/21 (33%)
**Clinical outcome**		
Discharged	32/32 (100%)	17/21 (81%)
Died	0	4/21 (19%)
